# Supplementary material for: Patterns of Intron Gain and Loss in Fungi
Source: PLoS Biol. 2004 Nov 30;2(12):e422. doi: 10.1371/journal.pbio.0020422 (PMC532390; doi:10.1371/journal.pbio.0020422)
Supplement: Table S1 — Also available at http://genes.mit.edu/NielsenEtAl/. (4.3 MB ZIP). [file pbio.0020422.st001.zip › NielsenEtAl/html/1288.html]

AN3460.1.NCU02402.1.MG03573.1.FG09838.1


```
 CLUSTAL W (1.82) Multiple Sequence Alignments - Introns Inserted


Sequence 1: NCU02402.1	1192 aa
Sequence 2: MG03573.1	1194 aa
Sequence 3: FG09838.1	1164 aa
Sequence 4: AN3460.1	1548 aa
Alignment Length: 1638 aa
Number Identitical Residues: 184 aa
Alignment Score (without introns) 13259


MG03573.1 	-MPKRTVNGLAGSPAHKRTRFEQDEDEVVDTAESSSARRNESVSSSEAPDIEVLEIA~LE
NCU02402.1	-MPARTL-------------FEAEAN-----AESSRKRQRPAPTSDDS--------E~AE
FG09838.1 	MAPTKRS------------RRDAEQD-----DEITDVQRARSNYGNGD--------A0RK
AN3460.1  	--------------------------------MSNQKRSQESQDFSDT---------~-D
          	                                  .  :   :   .             .

MG03573.1 	AAF~TDDDKQ----RK~RVRLSTPEDVKPIIPSHSSRRAPNPSNDDEEEDGEEEDFNMED
NCU02402.1	IAA0RPNANQ----AK~RARVDT------------ARHATPVDDDDVDVDDERG-----H
FG09838.1 	RAR~VTLDDE----RT~IEQP--------------PRREETPSDESDDDDDDRR------
AN3460.1  	HED~TSSDRSSLSLQT0-------------------RKRPRVSDFAEFHRSDHS------
          	         .: :  .                    *:    .:      .:.       

MG03573.1 	EGEGDLVAGTQYEMLRDKEFAHLQHEKEDDQRATQRLRSRPNIVG---QNHTAENGILLR
NCU02402.1	AGSPHSPPKTQYELMRDNNFEHLQHQDADDQRATQRLRFKPNQLG---DNAVADNGILES
FG09838.1 	--QRNSPPRTQYEMMRDDGFKHLKHADWDDQQATQKLAQRSTTLG---NNMVSESGIIES
AN3460.1  	--LAFSDIGGDTARATTSSTVDLEQDEEELELLATQIIQEKYSFGSDEPNAPSEHGILER
          	          :      .   .*:: . : :  : ::  .   .*:.. *  :: **:  

MG03573.1 	VECVNFMCHERLNVELGPLMNFIVGENGSGKSAILTAITVCLGGKASSTNRAGSLKALVK
NCU02402.1	ITCINFMCHTRLHCELGPLLNFIVGENGSGKSAILTAITLCLGGKASSTNRGGSLKSFVK
FG09838.1 	ITCFNFMCHERLHVDLGPLINFIVGENGSGKSAVLTALTLCLGGKASDTNRGGSLKSFVK
AN3460.1  	VECYNFMCHDHFQVELGPLINFIVGKNGSGKSAVLTAITLCLGGKASTTNRGQSLKSFIK
          	: * ***** ::: :****:*****:*******:***:*:******* ***. ***:::*

MG03573.1 	SGQQ~QAILSVTIKNEGLDAFQHDIYGDSITVERHFSTTGSSGFKVKSKSGRIIGTKKAL
NCU02402.1	EGTE~KAVLIVKIKNQGQDAYRHELYGDSIIVERHFSKSGSSSFKVKTATGQIVSTKKQE
FG09838.1 	EGTE~HGSLVVKIKNAGSDAYQPDIYGETIIVERHFSKSGSSGFKIKSANGRIISTKKQE
AN3460.1  	EGKE2SATIIVRIKNQGDGAYLPDDLGKSIIVERHFSKSGASSFKIKADNGRIFSTKRTE
          	.* :  . : * *** * .*:  :  *.:* ******.:*:*.**:*: .*:*..**:  

MG03573.1 	VEEIVEYFCLQVDNPLNVLSQDQARSFLNSASDSEKYQFFIMGVQLEQLDNDYRLVNEFV
NCU02402.1	VEEIVEYYALQVDNPLNVLSQDNARQFLNSSTKQQKYKFFIEGVQLQQLDNDYRLIAESL
FG09838.1 	VDEISEWYALQIGNPLTVLSQDNARQFLNAATPAQKYKYFVSGVQLEQLDNDYKMSQDTL
AN3460.1  	LDAIIDHFTLQFENPMNVLSQDMARQFLSSSSPAEKYKFFVKGVQLEQLDQDYRLIEEYG
          	:: * : : **. **:.***** **.**.:::  :**::*: ****:***:**::  :  

MG03573.1 	ESARSKIPEQEERVVLTKKKYEEAQKLHESMKQNEELRRKRRLYAQQLAWAQVQDQEK~I
NCU02402.1	ELMESKIPDQEERVKAAAEELKRAKSFKDAMDGNRKLRAKQHQLRSQMCWLQVVQEEA~K
FG09838.1 	EKTSLLRDDLDSKIEQVKKEMEEAKRLSETAQKNKTMREKARHYRNQLVWYQVVEQEQ~S
AN3460.1  	DQIEEKIKSKQQDVSVLKNRRDAAERKLEMSDQQENLRERQRKLRRQAAWAQVEEQER0I
          	:       . :. :    :. . *:   :  . :. :* : :    *  * ** ::*   

MG03573.1 	LEGRHDKVRDVNDRIRAQELEIERKSAAFDAADRTYERAVAEAERMAEEEPEAAAMTDAA
NCU02402.1	LTKLDEKIAQLADQMAEVDRQRNEKGVDLERVENQIRAFQQRLEEAIQRKTEFEEQVDEG
FG09838.1 	LAEYEKDLVRRAQTIIEKEQYCESTTEELRLTEEKLEQRRQTKEELEQDRGFYEESIAKA
AN3460.1  	RDSLIAEISSLDSKISEVEAEVARCDAAIREVEAEAITAAQYCREASAKVDNAQNERNDI
          	      .:    . :   :         :  .:          ..               

MG03573.1 	QEKFDAARRELERIR~QDERDAHTQLKHHDKALKANAASIEQEKKIVAGSTGEEVTRNRD
NCU02402.1	RKKAQAIRDELAQIQ~ADERAAHQNLRSAATTVKDFEEKVAAEEKRLEEATGEALLSKTR
FG09838.1 	TEAHREAAKDESELL~MEERDAHQRLKTIKDDIKACKSKIQEEERRLSQSTGNARSEKDN
AN3460.1  	EARWNEALNERHELQ0AEQRRIREHVREANARIQQLQHQVDEETRRLADLHGGGYSRKLD
          	         :  .:   ::*  : .::     ::    .:  * : :    *     :  

MG03573.1 	RLAAEKDRLEQIRERLNENAARGPGLEQLRDDAKNEQIKMHRAAEAKQQEIRAQEGKIRD
NCU02402.1	ELEKAKEYVTEVEAKISKAKEDEKDLLNKRDEAQKARDAKAVECSLKRDEITVAEQELRT
FG09838.1 	ELEQARDKESLLRQQMEEIHNNLPDLQTRLSDAEHELKKLSYSKDIKRKDIVSIEQQVRE
AN3460.1  	ELERAKQDAMEVRKQIDELEQNASQLSDDIRAAESQEKAAYQPVAQARRDLEEANSLLHN
          	.*   ::    :. ::.:       *      *:             : ::   :  :: 

MG03573.1 	LERNDRSPLDGYEPRVPELLRQIASDSGYREKPIGPMGSLIRLHKPEWSSLLEKTLGQQL
NCU02402.1	SEKDQGSIYAAYEPKLPELLKMIEKETRFQKKPVGPLGAHIQLLKPEWSLILEKTFGTAL
FG09838.1 	LKATTGGRFDGYDRDIRDVVSAIENEQGWEQKPVGPIGAHIRLSKPEWSGILERTLGEGL
AN3460.1  	LNREGSGRNSGFPERMSALLKAIQQNRSFTETPVGPIGNFVTLLKPEWSSILESSFGATL
          	 :    .   .:   :  ::  * .:  : :.*:**:*  : * ***** :** ::*  *

MG03573.1 	NAFVVTSKADQQRLQTTMRRLGI~QRCP-IAIGNRAPLN~TADKEPDTEFDTILRVLKFD
NCU02402.1	NAFIVQSMAEQKLLQGLMNKLNI~RQCP-VFIGNRHPLN~TDGKEPDPSFDTILRVLKID
FG09838.1 	NAFVVRSKSDQTRLSNLMRRLRL~KKQPPIYIAYGGKID~TSSQEPDPAFDTILRVLQFD
AN3460.1  	NGFIVTSKRDQSILSEIMHRVNC2TSQINNSIRFYEFFR2N----------------KFD
          	*.*:* *  :*  *.  *.::          *     :  .                ::*

MG03573.1 	DDLIRNQLIISGSIEQVILIPDRKAAEKAMFDGPPPRNVKACLSFNERTKSRGYRLTARQ
NCU02402.1	NMMVRDQLIINHMIEQVILIPQRTKAEDIMFSGARPRNVKACLSFHDKKRDEGLRLVVNN
FG09838.1 	DDIVRSQLIIGNQIEKIILIRDREEAQKVMVEGVPPRNVGACLCFHDGQNKRGWGLRLTN
AN3460.1  	NELVRRQLVINHGIEQNLLIENLEEASSVLFDGERPRNAKRCFCINKSDRRRGILLSYSR
          	: ::* **:*.  **: :** :   *.. :..*  ***.  *:.::.  . .*  *   .

MG03573.1 	GGQNLSTSQVNPNPGTKPRMKADAHAQIA~QEREYLAQLKDEYTRLCQKRDDQRRKVQQC
NCU02402.1	SG-GMSTSPVPPLRNQRPRMKADIGSRLA~YQKETLRQLELEYSVLDREHRQLQQEVQKI
FG09838.1 	RNGSMATSPVQPYT-MRPRMQTDAGQQVR~IQEDNLKHLGLEMAEINRKERQAQQAVQRC
AN3460.1  	NG-EPSQAPVSVYS-GSPRMKSDRDSQIR2VQREAVANLRQVLNAREEELRSAQSHLTRC
          	 .   : : *       ***::*   ::   :.: : :*        .:  . :  : : 

MG03573.1 	EQELQQQRQTKTELHREERQCEASILGIERDLDQFEGYDARLQGLEEARARLQEEFETVG
NCU02402.1	ISDLTKLQRDKKTLDSELRHARVQVEQVQYRLDEYEGGDSRLTGLKAELAAAKEKEEACG
FG09838.1 	RTELDNQRKDNRKLEGDLRRTQADIERVQMELDAFEGVDGRLNILRAELESRRSDEESLG
AN3460.1  	RQAFERNERKKNFLVIESQRKDDRIEELEESLQKEGSQDGDLEILQASLREAQEEKLTHE
          	   : . .: :  *  : ::    :  ::  *:   . *. *  *.      :..  :  

MG03573.1 	RQYGELRAIMDEQNKKVQALHAELAKEKATRDQFMARRGRAMDKIKGYADGRAMELSQKN
NCU02402.1	LQYGNLRIRKDEKNQASTEAQAQLTEIRKESEKYEREVKKLQEKKARLEDVRKINLTEVN
FG09838.1 	NQYGEMALSKRDLKAKTEKARLKLEEARNNQEEFEGKVQKASQKIATAENMRRVAVAKKN
AN3460.1  	GSLDDAANAMTEMMQNLKRIKKELATKDAEIARLKDELRVAESEQYLIADKRRKRIGEKN
          	 . .:      :        : :*        .   .     .:     : *   : : *

MG03573.1 	AAMEEMEKLKDDLRRAERKVGEQETIVQEFTTEASKIFP--ERVFVPEGETHQAVQKKLD
NCU02402.1	EAHASFEIFKGDKEEAEKEREKGAEDVATFTKQIIEALGSEERAHVNPTDKYEELEKQYQ
FG09838.1 	DAFEQVDIEKNERRRVEEKREHKAIEVAQFIEQATEAVP--ERVHIPEDETYDSIEQKYT
AN3460.1  	AAVELVDDTNRRRARMKQKRDSADADVLEYISKASVISE---RVEIDEGETPATLDRKLE
          	 *   .:  :    . :.:       *  :  :         *. :   :.   ::::  

MG03573.1 	IVKKQLDDRNKR~IGKTEAQVEAEAATAKKNFEHASKS~LKGSLQVIK~QLKRTMALRLE
NCU02402.1	SIQNQLEKERRK~RGMTDEEVYANLTRAKETYDDAKRS~LEGIKTVNS~RLRRTLTIRLE
FG09838.1 	KLREQLKQREAR~IGATDDQIFDRSREAEDRYESVQRQ~TKDLDDTIA~CLKRAIHSRLY
AN3460.1  	RVTRDMVTYSRE2LGGSREEIRAEADTAIKAHQQALKQ0VLKASLNHR1ISIRQALNQHV
          	 : .::     .  * :  ::  .   * . .: . :.              *    :  

MG03573.1 	KWRLFQRYISA-----------------------------------------~SARTNFI
NCU02402.1	KWRKFQRYISS-----------------------------------------~QSRANFI
FG09838.1 	MWRQFQRQISA-----------------------------------------~RIRIQFN
AN3460.1  	LTSQVEPDITKDSSEGRGARTLSGGEKSFSQVCLLLALWEAMGSPIRCLDEL2KMAIDML
          	    .:  *: .::.. .: : :....: :. .   :  .: .:.  . ..      :: 

MG03573.1 	~YLLSERGYRGQLLLDHVNRKLQIQVEPDATKKNG---------------~-----~---
NCU02402.1	~YLLSERGFRGKLLLDHERKALDLVVEPDKTEKRA---------------~-----~---
FG09838.1 	~YLLSERGFRGKIDLDHKNRKVHVQIEPDETRKSS---------------~-----~---
AN3460.1  	0MYAARRSVGRQFILITPGSRAEISLAPDVMVKEYGLTDFIQDKADWRIG1HCTLK0NLG
          	    :.*.   :: *       .: : **   *  . :.  ...:.   .  .: . . .

MG03573.1 	--QGRNTKTLFWW~R---EVLFFHLPVAGNMG-~--------------FHG2AISTNML0
NCU02402.1	--AGRNTKTLSGG~EKSFSSICLLLSIWEAMGS~PLRCLDEFDVFMDNVNR~AISTNML~
FG09838.1 	--SGRNTKTLSGG~EKSFSSICMLLSVWEAIGS~PIRCLDEFDVFMDNVNR~AISTNML0
AN3460.1  	DWTTADFVYLVDN1PKWKEQLKLPAKDGRPQTE0DVTATKGLEFEDFYIKR~ELMMGIF~
          	.    :   *     .  . : :         .    . .  .     .:   :  .:: 

MG03573.1 	VS-------------------------------------------AARRSVSRQYIMITP
NCU02402.1	VSFIDDGNDGDKQWYIHEPL------------------LTWPQITAARRSVNRQYIFITP
FG09838.1 	VD-------------------------------------------VARRSVSRQYILITP
AN3460.1  	EAGFEKPSPIQEETIPVALTGRDILARAKNGTGKTAAFVIPTLERINPKSTKTQALILVP
          	    .. .  ...       . .  : :...:..:::    .      :*.. * :::.*

MG03573.1 	N------------------------------AIEGRAKVDKDVKIHR2MTDPRQRTLAGP
NCU02402.1	N------------------------------AIEGRNTLDKDVKIIR2LTDPRQRTLADY
FG09838.1 	N------------------------------AIEGRARLDKDVKIIR2LTDPRQRTLDDF
AN3460.1  	TRELALQTSHVCKTLGKHLGINVMVTTGGTGLMDDIIRLNDAVHILV~GTPGRVLDLASK
          	. . : .::  ..: ..  . .   ::..:. ::.   ::. *:*    *  *   * . 

MG03573.1 	QAR-----------------------------------------------------~---
NCU02402.1	--------------------------------------------------------~---
FG09838.1 	R-------------------------------------------------------~---
AN3460.1  	GVADLSECPTFVMDEADKLLSPEFTPVIEQLLSFHPKDRQVMLFSATFPMIVKSFK0INQ
          	   . :...:   ..:..  :.. :.  ..  :  ... .    ::: .   .: .  ..

MG03573.1 	------------------------------------------------------------
NCU02402.1	------------------------------------------------------------
FG09838.1 	------------------------------------------------------------
AN3460.1  	SIIFCNSTNRVELLAKKITELGYSCFYSHARMLQQHRNRVFHDFRNGVCRNLVCSDLLTR
          	:   ..::.  .  :.. :. . :.  : :   ..  .    .  .. . .  .:.  : 

MG03573.1 	------------------------------------------------------------
NCU02402.1	------------------------------------------------------------
FG09838.1 	------------------------------------------------------------
AN3460.1  	GIDIQAVNVVINFDFPKNAETYLHRIGRSGRFGHLGLAINLINWDDRFNLYKIEQELGTE
          	. . .: .   . . ...:.:     . :.  .  . : .  . ..  .  . ... .:.

MG03573.1 	------------------------------------------------------------
NCU02402.1	------------------------------------------------------------
FG09838.1 	------------------------------------------------------------
AN3460.1  	IQPIPQNIDKKLYVYDSPDTIPRPISNPPQQSQGATAASNPSDRRHNHHTNGGHYQSKGR
          	 .. ... ...    .:..: . . :.....:..:::::..:.   .  :...  .:.. 

MG03573.1 	----------------------------------------
NCU02402.1	----------------------------------------
FG09838.1 	----------------------------------------
AN3460.1  	GPYRGGRGQGQRRNMQNDGTKFNAAQGQTGGKAQPPAQVS
          	..  .. ....  . ....:. .::...:...:...:. :
```
